# Response to a Comment on “Scar‐Degrading Endothelial Cells as a Treatment for Advanced Liver Fibrosis”

**DOI:** 10.1002/advs.202301330

**Published:** 2023-04-29

**Authors:** Peng Zhao, Tian Sun, Cheng Lyu, Yanan Du

**Affiliations:** ^1^ Department of Biomedical Engineering School of Medicine Tsinghua‐Peking Center for Life Sciences Tsinghua University Beijing 100084 China

David Dolivo et al. recently shared their views regarding our study on in vitro primed endothelial cells, which were capable of degrading scars. The authors proposed that the current treatment strategy for fibrosis should be broadened to involve other cell types, stimuli, elements of the extracellular matrix (ECM) and targeted organ types. Transcriptomic profiles have been specified as an effective resource for predicting cells with the capability to degrade the ECM. The authors were also concerned if the ECM‐degradation therapy for fibrosis would be accomplished in other organs (e.g., cardiac fibrosis), which did not possess the same regenerative capabilities as the liver. Overall, we found these comments quite inspiring for future endeavors in cell‐degradation therapy in the treatment of fibrosis in multiple organs.

As liver fibrosis advances, collagen I is the most prominent ECM component.^[^ ^1^ ^]^ We centered our study on collagen I for construction of a cell‐mediated ECM‐degradation screening system (CEDSS),^[^ ^2^ ^]^ whereas other ECM components such as fibronectin, elastin, and collagen III were absent. In the future, CEDSS needs to be further improved to tailor the concentration of collagen I, supplement with other ECM components, and include multiple ECM crosslinking schemes^[^ ^3^ ^]^ (e.g., lysyl oxidases, transglutaminases, and advanced glycation end products) according to the characteristics of pathological ECM in different fibrotic organs. This would be more bionic and effective in screening fibrotic ECM degrading cells for specialized organs.

Utilizing cell type‐specific transcriptomic profiles to predict cells with the capacity to break down the extracellular matrix (ECM) is a noteworthy concept. For instance, by assessing the expression of proteases related to the ECM degradation of various cell types, we can identify potential ECM‐degrading cells and related regulatory mechanisms. Nevertheless, omics data of transcriptomic profiles only reveal the expression of proteases at the gene level, thus the function of ECM degradation of the cells should still be determined and validated by functional assays based on CEDSS.

Besides, in vitro stimulation approaches to prime cells should not be limited to chemical compounds and cytokines. Other stimuli such as physical stimuli (e.g., temperature,^[^ ^4^ ^]^ pH,^[^ ^5^ ^]^ magnetic field^[^ ^6^ ^]^), mechanical stimuli (e.g., substrate viscoelasticity,^[^ ^7^ ^]^ pressure,^[^ ^8^ ^]^ shear force,^[^ ^9^ ^]^ stretching force^[^ ^10^ ^]^) and gene editing (e.g., activation^[^ ^11^ ^]^ or inhibition^[^ ^12^ ^]^ of a particular gene pathway) can be also employed for in vitro cell priming.

The extraordinary regenerative capabilities of the liver^[^ ^13^ ^]^ could be a factor in the success of this antifibrosis strategy, raising doubts about its applicability to the treatment of fibrosis in other organs with limited regenerative capacity. It is definitely worthwhile to try on cardiac, kidney, and skin fibrosis, etc. A potential improvement would be to cotransplant stem cells^[^ ^14^ ^]^ along with or after the ECM‐degrading cells, which could differentiate into or promote proliferation of the parenchymal cells^[^ ^15^ ^]^ after the ECM degradation.

Furthermore, tissue‐specific cell‐based ECM degradation therapy would be a necessity for fibrosis treatment in different organs, owing to the disparity in ECM components, concentrations and crosslinking. Meanwhile, controlling the degradation degree of the pathological ECM is a key safety concern that must be taken into account when performing clinical treatment, as it is necessary to ensure that any excess ECM is degraded without damaging the function of the tissue. To avoid ECM breakdown in nonfibrotic organs and tissues, targeted delivery of cells should be imperative, which can be accomplished by modifying the cell membrane with organ‐targeting peptides^[^ ^2^ ^]^ or employing a mechanoresponsive cell system (MRCS).^[^ ^16^ ^]^

## Conflict of Interest

The authors declare no conflict of interest.

## Author Contributions

P.Z. and T.S. contributed equally to this work. All authors wrote the manuscript and approved the final manuscript.
